# Competencies for One Health Field Epidemiology (COHFE)—a framework to train the epidemiology workforce

**DOI:** 10.1186/s42522-025-00135-x

**Published:** 2025-03-30

**Authors:** Marion Muehlen, Navneet Dhand, Heather Simmons, Stacie Dunkle, Christine Budke, Ahmed Zaghloul, David Castellan, Silvia D’Albenzio, Ravi Dissanayake, Jessica Cargill, Stephen Leshan Koyie, Julio Pinto, Barbara Alessandrini, Karl Schenkel

**Affiliations:** 1 World Health Organization, 20, Avenue Appia, CH-1211 Geneva 27, Switzerland; 2 Food and Agriculture Organization of the United Nations, Rome, Italy; 3 World Organisation for Animal Health, Paris, France

**Keywords:** Field epidemiology, One Health, Competency, Curriculum, Framework, Capacity development, Workforce

## Abstract

**Background:**

Field epidemiologists play a crucial role in addressing the complex challenges posed by emerging infectious diseases, transboundary animal diseases, and antimicrobial resistance. Despite the interdisciplinary nature of these issues, traditional field epidemiology training programs are often narrowly focused on specific sectors. To effectively confront these evolving challenges, it is imperative to equip field epidemiologists with the skills to adopt the One Health approach. However, there are neither globally accepted One Health competencies for guiding field epidemiology training programs nor standardized curricular guidance for program managers. Recognizing this gap, three international organizations joined forces to develop the Competencies for One Health Field Epidemiology framework.

**Methods:**

A desktop review was conducted of the existing frontline, intermediate, and advanced field epidemiology training program curricula. Knowledge, skills, and competency (KSC) statements for frontline, intermediate and advanced levels were then defined and grouped into domains and subdomains by thematic area. An international Technical Advisory Group of 59 experts from the animal, environment, and human health sectors was convened to review the proposed statements. The framework was revised based on their feedback. KSC statements were classified into core and optional, and a prioritization tool was developed to assist countries in selecting optional KSC statements based on their specific requirements.

**Results:**

The competency framework was developed and comprises KSC statements needed for field epidemiologists to successfully apply the One Health approach across the human, animal, and environment health sectors. These KSC statements are stratified by frontline, intermediate, and advanced training levels and are further categorized as core and optional; sector-specific KSC statements are also identified.

**Conclusions:**

This innovative framework emerged from a multisectoral, collaborative, inclusive, and iterative process involving international animal, human, and environment health and field epidemiology training experts. Countries and regions can also use the framework to establish new, comprehensive One Health field epidemiology training programs or upgrade existing programs to incorporate the One Health approach. This framework is anticipated to pave the way for a more holistic approach to training the global community of field epidemiologists in all health sectors to meet the demands of our evolving health landscape.

**Supplementary Information:**

The online version contains supplementary material available at 10.1186/s42522-025-00135-x.

## Background

Over 30 emerging infectious diseases (EIDs) have been detected in the past three decades [1]. Notably, the majority of these EIDs trace their origins to animals, often spilling over from wildlife or domesticated animals to humans under conducive conditions [1, 2]. For example, the investigation into the initial transmission of SARS-CoV-2 that caused the COVID-19 pandemic has explored various potential sources, including the handling of wild animals [3]. Additionally, the interconnectedness of the animal-human-ecosystem and environmental changes have played a significant role in the COVID-19 pandemic. For instance, alterations in global bat diversity underscore a potential link between climate change and the emergence of both SARS-CoV-1 and SARS-CoV-2 [4]. Clearly, EIDs exhibit a complex ecology, intertwining human, animal, and environment health systems and sectors.

To address EIDs effectively, establishing a One Health (OH) workforce involving various sectors and across various levels is imperative. Such a workforce would be able to operate seamlessly across diverse health sectors, fostering joint sector investigations, communication, coordination, and collaboration. However, conventional field epidemiology training for health professionals tends to be specialized within a single sector—be it human public health, animal health, or environment health. Thus, a paradigm shift is needed to cultivate a multidisciplinary approach capable of comprehensively tackling the intricate challenges posed by EIDs. The OH advisory group for Quadripartite organizations, the OH High-Level Expert Panel (OHHLEP), has emphasized that the health of animals, humans, and the environment are closely linked and defined a new OH definition: “OH is an integrated, unifying approach that aims to sustainably balance and optimize the health of people, animals and ecosystems. It recognizes the health of humans, domestic and wild animals, plants, and the wider environment (including ecosystems) are closely linked and inter-dependent…”[5]. To ensure the operationalization and implementation of the OH approach for EID prevention, preparedness, outbreak investigation, integrated surveillance, response, and recovery, it is critical to train field epidemiologists in the OH approach. The Quadripartite OH Joint Plan of Action [6] has identified a change pathway for OH implementation that includes scaling up capacity development at the regional and national levels and has prioritized strengthening collaborative, multisectoral competencies to support implementing OH systems.

Field epidemiology training can strengthen preparedness by enhancing countries' capability for early detection and prompt response to infectious diseases. Initially started by the US Centers for Disease Control and Prevention (US CDC) in the 1950s, Field Epidemiology Training Programs (FETPs) are now running in more than a hundred countries, training public health professionals in tackling disease outbreaks and conducting surveillance [7–9]. A key feature of these programs is that FETP fellows are expected to spend > 75% of their training time in the field under the guidance of mentors [10]. FETP training is typically offered at three levels: frontline (3–4 months), intermediate (9–12 months), and advanced (2 years). These programs are generally hosted by national ministries of health or public health institutes and, for advanced programs, often associated with academic institutions. Field Epidemiology Training Programs for Veterinarians (FETPVs) have also been implemented in several countries supported by the Food and Agriculture Organization of the United Nations (FAO) and follow the FETP model to strengthen the capacity of Veterinary Services to tackle infectious diseases [11–14]. These programs are also offered at three levels and are typically housed within agriculture or animal health ministries. Similarly, field epidemiology and laboratory training programs, with or without veterinary programs, have also been established to train candidates in epidemiology and laboratory skills.

The OH community recognizes the need for well-trained field epidemiologists to strengthen capabilities in disease surveillance, epidemiological investigations, and outbreak response. Training of veterinarians in field epidemiology is also considered a priority by FAO and the World Organisation for Animal Health (WOAH). The presence of an FETP in a country has also been included as an indicator in the World Health Organization (WHO) Joint External Evaluation tool since 2016, further motivating countries to develop and/or strengthen their capacity in these areas. Although organizations such as the US-CDC, WHO, FAO, WOAH, the European Centre for Disease Prevention and Control (ECDC), and the Training Programs in Epidemiology and Public Health Interventions Network (TEPHINET) have established relevant field training programs with curricula and competencies, there are no internationally accepted OH competencies for field epidemiologists, nor are there standardized curricular guidelines for field epidemiology training programs with a OH focus or continuing education requirements for program graduates.

Competency-based training is an educational approach that focuses on developing specific skills, knowledge, and abilities required for effective performance in a particular profession. Unlike traditional educational models that rely heavily on knowledge acquisition and time-based progression, competency-based training places emphasis on learners mastering clearly defined competencies under the guidance of a mentor. These competencies are the measurable and observable abilities that individuals should acquire to perform tasks successfully. This approach is widely adopted in various sectors, including healthcare and veterinary training, as it aligns training outcomes more closely with real-world performance expectations. For example, the WHO has developed the Global Framework for Universal Health Coverage to support countries in realizing the vision of better health [15] and WOAH has developed competencies for graduating veterinarians [16]. Similarly, the Competency-Based Veterinary Education framework has been developed by the American Association of Veterinary Medical Colleges to ensure the delivery of high-quality animal healthcare services [17]. FAO developed field epidemiology competencies for frontline and intermediate-level field epidemiology [12]. These competencies are used in developing FETPV frontline programs in Asia, the Middle East, and Africa. Developing competency-based training for OH field epidemiology is a crucial step towards enhancing field epidemiologists' overall competence and preparedness, contributing to the well-being of animals, humans, and the environment.

Realizing the gap in the availability of globally recognized OH competencies for field epidemiologists, FAO, WHO, and WOAH collaborated to develop the Competencies for OH Field Epidemiology (COHFE) framework (https://www.who.int/initiatives/cohfe-framework). It includes statements to provide an understanding of the essential knowledge, skills, and competencies (KSC) that field epidemiologists will need to successfully apply the OH approach across the human, animal, and environment health sectors at the frontline, intermediate, and advanced levels of training. This article summarizes the approach adopted for developing these KSC statements that countries, regions, and field epidemiology training programs could use to train their OH workforce.

## Methodology

**Fig. 1:**
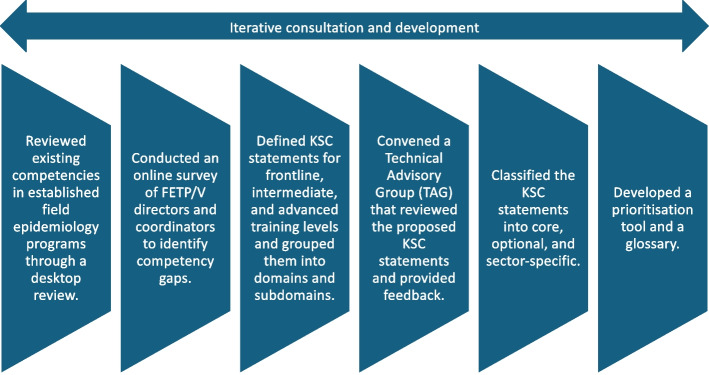
The approach used to develop competencies, curricula, and supplemental manuals for field epidemiology training using the One Health approach

FAO, WHO, and WOAH established a core technical team of about a dozen veterinary and human medical science experts, including pedagogical and methodological experts, who met virtually weekly in 2021–2022 to draft the competency framework. The Core team started by defining basic terms and taxonomy such as knowledge, competency, skill, domain, subdomain, frontline, intermediate and advanced levels of training (Table 1). The overall approach used by the team is outlined in Fig. 1.

**Table 1:** The definitions used in developing the COHFE framework

**Frontline**: A 3–4 month mentored in-service applied training programme for health workers to strengthen epidemiologic capacity at the community to district level. It aims at improving competencies to conduct data collection, disease monitoring, and support investigation and response to health events, across the One Health spectrum.
**Intermediate**: A 6–9 month mentored in-service or fulltime applied training programme for health workers who provide epidemiologic services usually at the district to provincial levels. It includes additional training in surveillance, data analysis and interpretation, and management of investigations and responses to health events, across the One Health spectrum.
**Advanced**: A two-year mentored fulltime intensive training programme for experienced health workers to prepare them for applied epidemiology leadership roles at provincial and national levels. It includes advanced training in designing and managing surveillance programmes, complex epidemiologic methods and management of investigations and responses to health events, across the One Health spectrum.
**Domain:** A broad topic or subject area that is divided into subdomains.
**Subdomain:** A narrower topic or subject area than a domain; subdomains consist of knowledge, skills, and competencies.
**Knowledge**: Assimilation of information through learning. Knowledge is the body of facts, principles, theories, and practices related to a field of work or study. It is described as theoretical and factual.
**Skills**: Ability to apply knowledge and to complete tasks and solve problems. Skills are described as cognitive (involving the use of logical, intuitive, and creative thinking) or practical (involving manual dexterity and the use of methods, materials, tools, and instruments).
**Competency**: Proven ability to apply knowledge, skills and personal, social and methodological abilities (attitudes and behaviours), in work or study situations and in professional and personal development in terms of responsibility and autonomy. It is not limited to cognitive elements (involving the use of theory, concepts, or knowledge), as it also requires the use of interpersonal skills (e.g., social or organizational skills) and ethical values where relevant. A core competency is the minimum level of competency expected to be achieved by the participants in a training programme.
**Core:** A required knowledge, skill or competency for a specific level of training (Frontline, Intermediate or Advanced) for One Health field epidemiologists.
**Optional:** A knowledge, skill, or competency that a country programme can choose to include in their Frontline, Intermediate or Advanced programmes based on a country needs assessment but which is not considered a required core competency for One Health field epidemiologists.
**Higher order competency:** An overarching One Health competency statement that summarizes at the subdomain level the more specific and detailed competencies in the Competencies for One.

### Desktop review

We performed a desktop review of existing frontline, intermediate, and advanced FETPs and FETPVs curricula (27 programmes), listed in Table S1 in Supplementary Materials. We also reviewed additional documents and guidelines to support the process, such as previous desktop reviews, evaluations, scientific publications, or frameworks for FETP/V programs. This enabled us to identify KSC statements suitable for the COHFE framework, some of which were adapted for use in the framework.

After reviewing competencies addressed by current FETP/Vs, we conducted an online survey of a broad geographical range of FETP/V program directors and coordinators to corroborate the findings and identify competency gaps from the desktop review. The detailed methodology and results of this online survey are described elsewhere (manuscript in preparation). The desktop review and the survey enabled us to finalize a list of competency domains and OH KSC statements.

### Defining OH Field Epidemiology KSC statements

After the review, we assigned a domain lead from the core technical team for each domain. The domain lead, in consultation with the core team, (a) defined KSC statements relevant to the domain, (b) consolidated and grouped statements into subdomains by thematic area, (c) specified if these were OH (cross-cutting) or sector-specific (human, animal, and environment health) statements, and (d) proposed the level (frontline, intermediate, or advanced) at which they should be applied.

We started by defining KSC for the frontline level. When defining KSC statements at intermediate and advanced levels of training, statements of the previous level were automatically included because trainees at higher levels were also assumed to require competencies identified for the lower levels. This meant that the KSC statements of the intermediate level also included the statements defined at the frontline level, and those defined at the advanced level included the KSC statements of the frontline and intermediate levels. The draft domain structure underwent two rounds of review by the core technical team before all domains were compiled into an initial draft COHFE framework.

### Technical Advisory Group (TAG) review

In August 2021, we convened an international TAG of 59 experts from the animal, environment, and human health sectors and all six WHO Regions to review the proposed KSC statements and suggest improvements. An inception meeting was held on 1 September 2021 to introduce the framework to the TAG, address questions, and explain the methodology of involving the TAG to finalize the competency framework.

**Fig. 2:**
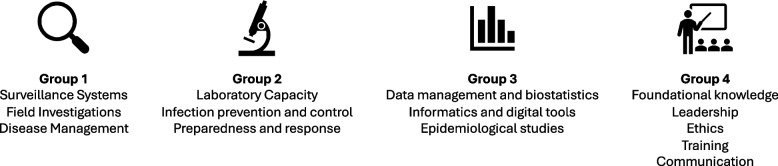
Subgroups in the Technical Advisory Group assigned various domains for review

TAG members were grouped into four working groups according to their expertise and preference, and each group was tasked with reviewing a subset of domains and subdomains and their related knowledge, skills, and competency statements in the draft COHFE framework (Fig. 2). Working group chairs and rapporteurs were selected to ensure maximum ownership of the review process by the participants and receive written feedback. Between September and October 2021, each of the four TAG working groups met virtually once weekly during a 4-week period to jointly review, discuss, and amend KSC statements where needed. Working group members had the opportunity to propose edits to the wording of the statements and, more importantly, to propose additional statements to ensure none had been missed by the technical team.

In addition, an online survey was deployed to ensure a systematic approach to collating and synthesizing each working group’s feedback. The online survey’s objectives were to assess if (a) experts considered KSC statements as adequate for a field epidemiologist working under a OH approach, (b) KSC statements had been assigned to the appropriate sector (OH, Human, Animal, or Environment Health), (c) if the statement corresponded appropriately to their proposed level of training (frontline, intermediate, and advanced), and (d) if the classification of knowledge, skills and competency statements was appropriate. The TAG working groups provided feedback, suggested edits, and proposed improvements via an online survey tool and through discussions during four virtual meetings. Besides the TAG review, the draft competency framework designed by the core technical team was also reviewed within FAO, WHO, and WOAH, expanding to their respective broader community of subject matter experts. Feedback from the organizations was received using the same online survey or via email.

After the TAG review, the technical core team addressed all comments and revised the structure and content of the competency framework. Comments and suggested edits were categorized according to whether they addressed an existing statement, proposed a new one, or were of a general nature. Each comment was either accepted or rejected or determined to require additional internal discussion. Results and replies were included in a spreadsheet for follow-up and accountability. The TAG and internal reviewers were informed of the review's outcome in a subsequent feedback meeting held in 2022. Besides changes to the existing competency domains, three key outcomes of the TAG review were the development of a new Ecosystem Health Domain (Table 2), a Systems Thinking subdomain, and confirmation of a need to develop a glossary with definitions agreed upon across sectors (Table 3).

**Table 2:** Developing the new Ecosystem Health Domain based on the TAG feedback

The experts who reviewed the competency framework identified gaps in the environment sector competencies. Therefore, a subgroup of environment experts from the TAG was convened to address this gap. This group decided to develop a separate domain on Ecosystem Health with five subdomains. The group also proposed an initial set of competencies for each subdomain.
A separate but concurrent FAO initiative called the *Field Training Program for Wildlife, Ecosystems, Biodiversity and Environment (FTP-WEBE)* was being conducted to identify frontline competencies and training needs specifically for environment sector experts. It involved identifying domains, competencies and skills for Biodiversity and Ecosystems, Wildlife and One Health involving a group of about 20 professionals from multiple agencies, including UNEP, CBD, FAO, WHO, WOAH, academics and NGOs. Selected competencies from the FTP-WEBE framework relevant to field epidemiology were classified as sector-specific or One Health competencies and incorporated into the framework across the three training levels.

**Table 3:** Developing a One Health glossary

The TAG confirmed that agreed-upon definitions of terms would be crucial to understanding the competency framework, especially because some terms had sector-specific meanings. As a result, we compiled a One Health glossary while respecting the sector-specific definitions of certain terms.
Each domain lead reviewed the complete set of competency statements for their domain and identified the terms to include in the glossary. Duplicate terms were not excluded at the start, as there could be different definitions of the same term depending on the sector or domain. In the next step, several existing glossaries were consulted, and all suitable definitions were listed. If a description did not exist or was not appropriate, the domain lead proposed a suitable definition for the term within the context in which it was being used.
Each term was defined, respecting sector-specific definitions and references. In general, we applied existing technical terminology from the FAO, WHO and WOAH, e.g., in the areas of the (veterinary) health workforce, epidemiology, and surveillance, and from the glossary of the Tripartite Zoonosis guide, where applicable (https://www.who.int/initiatives/tripartite-zoonosis-guide). In addition, we used the UN Terminology database https://unterm.un.org/unterm2/en/.
Two team experts from the agencies not in the lead of the specific domain reviewed the terms and definitions proposed, e.g., if WOAH was leading the development of the Domain 7 glossary terms and definitions, a team member from FAO and WHO each would review the proposed terms and suggest edits. This review round allowed us to narrow down the proposed definitions for duplicate glossary terms and discard unsuitable or duplicate definitions.
The competency framework yielded 129 glossary entries, including a listing of references consulted for each term.

### Classifying KSC statements as core and optional

**Fig. 3:**
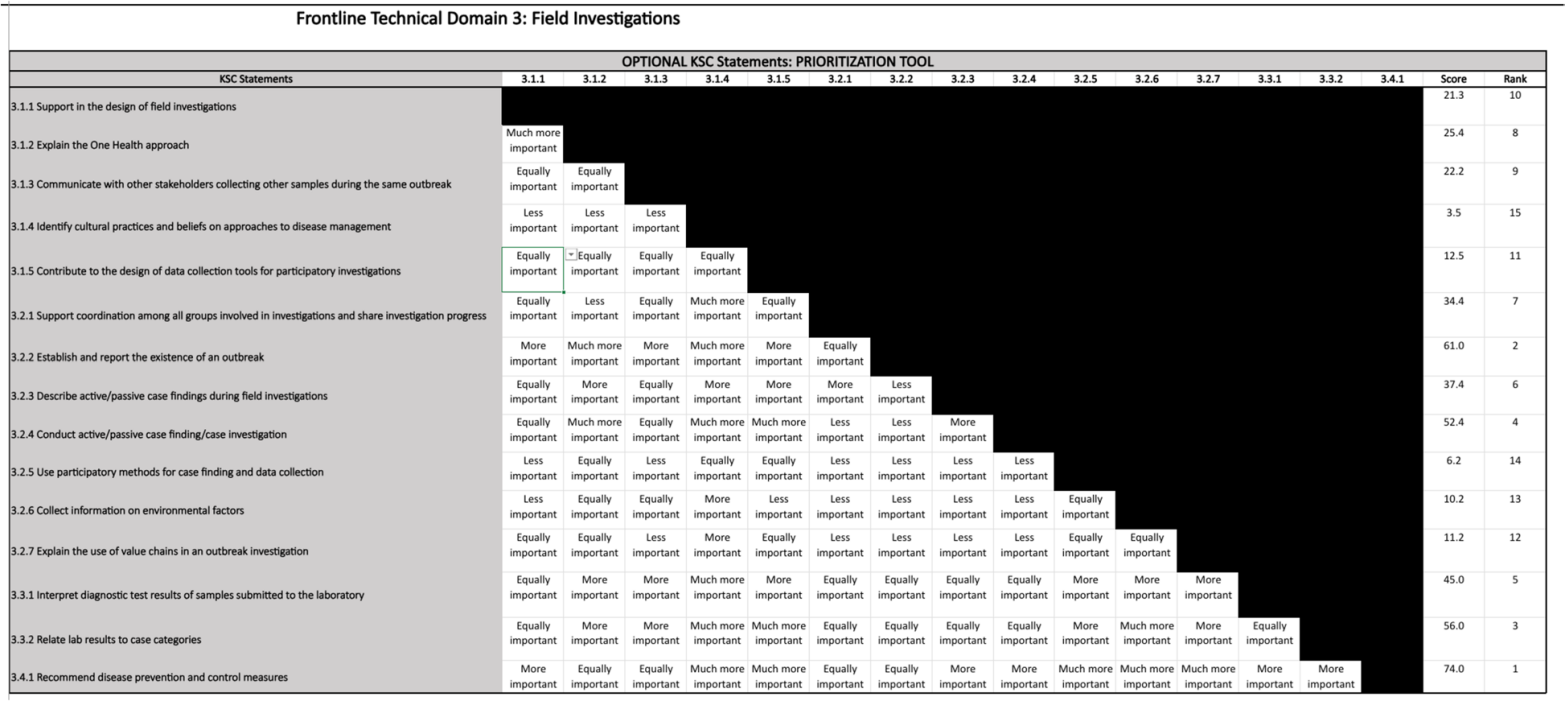
Example Ranking of Optional COHFE Framework Competencies for Domain 3 – Field Investigations

On completing the TAG review of the COHFE Framework, TAG members were requested to classify COHFE KSC statements as core or optional using a commercial survey tool for each training level (i.e., frontline, intermediate, and advanced). Core statements were considered essential for establishing a country-level OH FETP or for upgrading existing FETPs, FETPVs, or other sector-specific FETPs to include competencies for One Health. All statements were assessed at four cut-off levels based on responses from TAG members (100%, 90%, 80%, and 66.67% of TAG members considered the KSC statement core). Finally, KSC statements were considered core when at least 90% of the TAG survey respondents selected them. All sector-specific statements (i.e., human health, animal health, and the environment) were excluded from the survey and considered optional statements throughout the process. See Table 1 for the definitions of core and optional KSC statements. A comprehensive prioritization tool was later developed (adapted from the Competency Prioritization Tool, Public Health Foundation, http://www.phf.org/resourcestools/Documents/3-Step_Competency_Prioritization_Sequence.pdf, accessed 10/11/2021) to support countries in selecting optional KSC statements according to their requirements (see Table 4 and Fig. 3 for details).

**Table 4:** Developing a prioritization tool

We developed a Frontline One Health Competency Prioritization Tool to help identify the relative importance of the optional One Health KSC statements within the context of the country’s needs and strategic objectives. This tool owes much of its design and function to the Competency Prioritization Tool developed by the Public Health Foundation (https://www.phf.org/resourcestools/Documents/3-Step_Competency_Prioritization_Sequence.pdf), which utilizes the pairwise comparison methodology (tailored for use in this tool) to allow organizations to prioritize competencies in the Core Competencies for Public Health Professionals.
The tool is divided into 14 domains so that all optional One Health KSC statements within a domain may be compared. The core One Health statements for each domain are listed for reference, and each optional statement for a domain is located in a ranking table. The tool ranks each KSC statement using pairwise comparisons. To make each pairwise comparison, an expert or group of experts will assign a rating from a drop-down menu. A Likert-type scale was used as “much more important,” “more important,” “equally important,” “less important,” and “much less important”.
In the example presented in Fig. 3, a pairwise comparison ranking has been made for each of the Optional KSC statements in Domain 3: Field Investigations. The KCS statement ‘Explain the One Health approach’ was rated as "Much more Important" to the KSC statement ‘support in the design of field investigations’. In the next row, Competency ‘Communicate with other stakeholders collecting other samples during the same outbreak’ was rated as "Equally Important" to the KSC statement ‘support in the design of field investigations’ and so on. The pairwise ratings were used to calculate a score for each competency, along with a ranking. Therefore, program planners may choose to include KSC statements that were rated the highest and may not include those that were rated the lowest. Note that this tool is meant to assist in decision-making rather than being prescriptive: cutoffs for inclusion or exclusion of competencies should be made on a case-by-case basis, depending on the country’s needs and priorities.

## Results

### Desktop review

Table S1 lists the curricula for various FETP/V programs reviewed before developing KSC statements. Several additional documents, such as planning guides, technical reports, and guidelines, were also reviewed before defining KSC statements.

### Defining OH KSC statements

Based on the initial desktop review and the online survey, KSC statements were developed and allocated to one of the 76 subdomains within ten technical and four functional domains. The final draft competency framework consisted of 846 statements across all domains, sectors, and levels of training, of which 666 were regarded as OH statements and 180 sector-specific statements.

### TAG review

Major outcomes of the TAG consultation and internal review were recommendations to a) merge the domains “Data Management and Biostatistics” and “Informatics and Digital Tools,” b) create a new “Ecosystems Health” domain (Table 2), c) create a “Systems Thinking” subdomain under the “Foundational Knowledge" domain and d) create a glossary. In addition, over 1000 comments and suggested edits were collected electronically via the online survey, 233 of which came from the internal review and 781 from TAG members.

### Competency classification

Upon initial review, more than 300 field epidemiology KSC statements were identified for the frontline level (33%), with an additional 280 for the intermediate (30%) and 324 for the advanced levels (37%). After initial review by the TAG, 102 (31%) KSC statements were categorized as core using the 90% cutoff, with 69% categorized as optional. After this, the core team reviewed an additional 37 optional KSC statements from six domains (i.e., foundational knowledge, surveillance systems, data management, biostatistics and digital tools, ecosystem health, leadership and management, and ethics) to determine if they should be recategorized to a core competency. After recategorization, 19 (57%) of the 37 KSC statements were recategorized from an optional competency to a core competency in the COHFE Framework. Based on this recategorization, 119 (36%) field epidemiology KSC statements were identified as core for the frontline level, with an additional 214 (52%) for the intermediate and 361 (62%) for the advanced levels.

### The revised competency framework

Following the revision of the domains, subdomains, and KSC statements by the TAG and internal review team, the updated document comprised 1300 KSC statements across 76 subdomains within ten technical and four functional domains. Of these, 490 statements were frontline, 448 were intermediate, and 362 were advanced. This included 453 OH Core KSC statements (172 frontline, 151 intermediate, and 130 advanced) and 545 OH Optional statements (189 frontline, 195 intermediate, and 161 advanced. The counts of OH KSC statements for different domains and levels are summarised in Table 5 below, and the details for various subdomains are presented in Supplementary Table S1. For the intermediate and advanced levels, it shows the number of ‘additional’ KSC statements at that level compared to the previous level. For example, if there are three KSC statements at the frontline level, two at the intermediate level, and two at the advanced level, this represents a cumulative total of three statements for the frontline, five for the intermediate, and seven for the advanced levels of training.

**Table 5:** One Health knowledge, skills, and competency (KSC) statements for frontline (F), intermediate (I), and advanced (A) levels in the final COHFE framework

Domains	One Health (Core)				One Health (Optional)			
	F	I	A	Total	F	I	A	Total
1. Foundational knowledge and skills	6	10	8	24	6	10	5	21
2. Surveillance systems	10	8	10	28	15	4	7	26
3. Field investigations	15	10	13	38	15	6	10	31
4. Disease management	19	10	18	47	8	9	12	29
5. Laboratory capacity	8	11	11	30	9	10	8	27
6. Infection prevention and control, biosafety and biosecurity	15	12	11	38	12	5	10	27
7. Preparedness and response	17	21	30	68	15	20	28	63
8. Epidemiologic studies	10	10	21	41	13	9	8	30
9. Data management, biostatistics, and informatics	17	15	13	45	4	2	13	19
10. Ecosystem health	11	7	12	30	25	24	33	82
11. Leadership and management	7	9	13	29	31	21	15	67
12. Communication and community engagement	5	5	8	18	10	11	14	35
13. Training	1	0	3	4	24	25	22	71
14. Ethics	10	2	1	13	8	5	4	17
Total	151	130	172	453	195	161	189	545

## Discussion

Field epidemiology training has made significant progress in addressing human, animal, and environmental health individually, but a critical gap persists in understanding the competencies necessary for field epidemiologists to implement the OH approach. Recognizing this gap, FAO, WHO, and WOAH conducted this work to define OH and sector-specific KSC statements for field epidemiology based on a desk review of existing frameworks and expert consultation. The resulting COHFE framework encompasses KSC statements from the animal, human, and environment health sectors. It proposes transversal core KSC statements in field epidemiology that exemplify the OH approach across all three sectors. This innovative framework emerged from a collaborative, inclusive, and iterative process involving international experts from the animal, human, and environment sectors, practitioners, academia, and representatives from all six WHO regions. It will support the implementation of the Quadripartite Joint Plan of Action, specifically Action Track 1, on strengthening health systems and developing workforce capacities for OH.

The development of KSC statements for the domain of ecosystem health, by including several environment health experts, is a notable feature of the COHFE framework. Existing training programs have partly addressed OH contents and curricula but tend to focus on the animal-human interface and specifically address zoonotic transmission. With its less anthropocentric approach, the OHHLEP’s definition of OH emphasizes the interconnectedness of the animal, human, and environment sectors and the need for intersectoral systems thinking and linking. By including this domain, we have highlighted environment health's critical role in understanding and tackling emerging infectious disease threats and fully accounting for the Quadripartite’s OH definition. This sector has been neglected in designing and implementing field epidemiology training programs. Still, with the increasing realization of the role of wildlife and environment in the emergence and spread of infectious diseases [18–21], it has become increasingly evident that the field epidemiology workforce needs competencies in environment health and a better understanding of the environment sector. However, these KSC statements will need to be further refined after trialing and implementing the framework. In particular, integrating environment sector competencies with other domains should improve future iterations of the framework and reflect how the human and animal health sectors learn to coordinate, collaborate, and communicate with the environment sector.

This framework now provides the most representative and comprehensive list of KSC statements for OH field epidemiology training across the human, animal, and environment health sectors at the frontline, intermediate, and advanced levels of training. However, the comprehensiveness of the framework also means that the sheer number of statements included in the framework is extensive. This is partly due to the nature of OH training, as several sectors are involved, and partly due to the decisions made by the Core Team to be as inclusive as possible to secure buy-in from all sectors. Realizing that it would be difficult for existing FETP/Vs to guarantee the integration of these additional KSC statements within the timeframes of their existing programs and for their completion, we classified the KSC statements into core or optional. The feasibility of incorporating all core OH KSC statements and potentially prioritized optional KSC statements from the COHFE framework into these same timeframes, or whether additional time is required, should be evaluated during the trial implementation of the COHFE framework. Nevertheless, countries or training programs that consider setting up OH curricula from scratch may benefit from the comprehensiveness of the COHFE framework.

Additionally, to support programs in implementing the COHFE framework, a prioritization tool was developed to enable them to select optional OH KSC statements according to their needs. This tool, after validation, will ensure that the adaption of the COHFE framework will suit the context and needs of the implementing country while guaranteeing the inclusion of the KSC statements considered to be core by the TAG. This will provide rigor in future OH field epidemiology training programs while providing flexibility to countries and regions to tailor the framework to their needs. Note that the prioritization tool is only a proof of concept because it only exists at the frontline level and does not include sector-specific KSC statements. However, we plan to expand the scope and improve the tool's usability moving forward. Further, to support programs implementing the COHFE framework, a series of guidance documents on curriculum development, mentorship, learning evaluation and certification, and continuing education have been developed and will be publicly available in due course. The supporting guidance documents complement each other and the COHFE KSC statements framework and curricula guidance.

This competency framework is intended to provide guidance to both the existing and future field epidemiology programs in human, animal, and environment health sectors. It can also be used by similar in-service training programs in related sectors, such as food safety and laboratory training. Existing programs can use this framework to identify selected domains, subdomains, or KSC statements to be included in their training program to ensure that their program complies with the OH approach. They can use the accompanying prioritization tool to shortlist optional OH KSC statements for inclusion in the program at the frontline level. Alternatively, FETP/V programs can choose to extend the duration of training to ensure the acquisition of OH KSC statements in addition to their existing sector-specific KSC statements. Countries and regions can also use the framework and the associated guidance documents to establish new, comprehensive OH field epidemiology training programs. Existing materials are available to guide countries on the establishment and maintenance of new FETP (e.g., 11, https://www.tephinet.org/sites/default/files/content/resource/files/fetp_development_handbook_0.pdf).

The need for OH field epidemiology training cannot be overemphasized, with the burgeoning challenges of climate change, antimicrobial resistance, emerging infectious diseases, and transboundary animal diseases we are facing requiring multi-disciplinary approaches to tackle them. This was reflected in the increased interest from existing field epidemiology training programs and experts for implementing the COHFE framework within training programs during the development process. Nonetheless, the proposed competency framework will undergo a pilot implementation phase in different regions to test its usefulness, validity, feasibility, robustness, and flexibility. Regional or country pilots planned to be implemented in the future will guide countries in how best to prioritize their individual needs for curricula planning and development and, at the same time, conclude how to make the competency framework and curricula guidance more practicable and user-friendly. This includes developing web-based solutions to guide countries in making the best use of our OH KSC statements and how to best translate them into curricula based on countries’ needs. The governance arrangements for such a program would also need to be tested as it is imperative to actively engage institutions representing the three sectors in the joint management and coordination of the program, securing mentorship and supervision from the human, animal, and environment health sectors, preferably from professionals with a track record of multi-sectoral collaboration. Despite these challenges, the COHFE framework holds the potential to significantly enhance the capacity of field epidemiologists to address complex health challenges through a unified OH approach. FAO, WHO, and WOAH intend to keep the engagement in strengthening national capacities in field epidemiology, testing the framework in the field, developing a community of practice, and enriching the program with additional implementation supporting tools. The partnership will be extended to the United Nations Environment Programme.

## Conclusions

The profound impact of the COVID-19 pandemic has underscored the highly interconnected nature of human and animal health and our environmental systems. This needs to be reflected in training upcoming generations of field epidemiologists who will have to deal with the increasing effects of environmental factors on health security. Bringing together experts from multiple sectors and fostering systems thinking and -linking, we successfully developed a comprehensive competency framework for OH field epidemiology training that includes an entire domain on ecosystem health and a subdomain on Systems Thinking. We anticipate that this framework will pave the way for a more holistic approach to training the global community of field epidemiologists. However, for this approach to be successful, it is imperative that training programs in the animal, human, and environment health sectors collaborate to provide joint training for future field epidemiologists, enabling them to navigate comfortably within and across sectors, to shed light on the interconnected mechanisms that lie at the core of health security. Thus, this field epidemiology OH competency framework not only anticipates the evolving challenges posed by environmental and climate-related factors but also lays the groundwork for a new era of field epidemiology training that embraces the interdependence of human, animal, and environment health to prevent and respond to health threats. Only through united efforts across sectors can we fortify the capabilities of future field epidemiologists to safeguard global health in an era of increasing complexity and interconnectedness.

## Supplementary Information

Supplementary Material 1. Table S1. Curricula for field epidemiology training programs were consulted to draft the COHFE framework sorted by country. Table S2. The number of One Health knowledge, skills and competency (KSC) statements in various domains and subdomains for frontline (F), intermediate (I), and advanced (A) training programs in the final COHFE framework.

## Data Availability

The datasets of the current study are available from the corresponding author upon reasonable request.

## Abbreviations

COHFE: Competencies for One Health Field Epidemiology
COVID-19: Coronavirus disease 2019
DTRA: Defense Threat Reduction Agency
ECDC: European Centre for Disease Prevention and Control
EID: Emerging infectious diseases
FAO: Food and Agriculture Organization of the United Nations
FETP: Field Epidemiology Training Programs
FETPV: Field Epidemiology Training Programs for Veterinarians
KSC: Knowledge, skills, and competencies
OH: One Health
OHHLEP: One Health High-Level Expert Panel
SARS-CoV: Severe acute respiratory syndrome related coronavirus
TAG: Technical Advisory Group
TEPHINET: Training Programs in Epidemiology and Public Health Interventions Network
US CDC: US Centers for Disease Control and Prevention
WHO: World Health Organization
WOAH: World Organisation for Animal Health
