# Supplementary material for: Competencies for One Health Field Epidemiology (COHFE)—a framework to train the epidemiology workforce
Source: One Health Outlook. 2025 Mar 30;7:13. doi: 10.1186/s42522-025-00135-x (PMC11955133; doi:10.1186/s42522-025-00135-x)
Supplement: Supplementary file 1 — Supplementary Material 1. Table S1. Curricula for field epidemiology training programs were consulted to draft the COHFE framework sorted by country. Table S2. The number of One Health knowledge, skills and competency (KSC) statements in various domains and subdomains for frontline (F), intermediate (I), and advanced (A) training programs in the final COHFE framework. [file 42522_2025_135_MOESM1_ESM.docx]

*Table S1. Curricula for field epidemiology training programs were consulted for drafting the COHFE framework, sorted by country*.*

| **Country/**  **Organisation** | **Program name** | **Level** |
| --- | --- | --- |
| Australia | Australian National University | Advanced |
| Brazil | Brazil Field Epidemiology Training Program | Advanced |
| Cameroon | Cameroon Field Epidemiology Training Program | Advanced |
| Canada | Canada Field Epidemiology Program | Advanced |
| CDC | FETP-Frontline Curriculum Guide | Frontline |
| CDC | FETP Frontline Planning Guide | Frontline |
| Egypt | Egypt Field Epidemiology Training Program | Advanced |
| Ethiopia | Ethiopian Field Epidemiology Training Program (Laboratory) | Advanced |
| Ethiopia | Ethiopian Field Epidemiology Training Program | Advanced |
| European Union | European Centre for Disease Prevention and Control (ECDC) | Advanced |
| FAO | Field Epidemiology training program for veterinarians | Intermediate |
| FAO | In-Service Applied Veterinary Epidemiology Training | Frontline |
| Germany | German Field Epidemiology Training Programme | Advanced |
| Mediterranean region | Mediterranean and Black Sea Programme for Intervention Epidemiology Training | Advanced |
| Mexico | Mexico Field Epidemiology Training Program | Advanced |
| Mozambique | Mozambique Field Epidemiology and Laboratory Training Program | Advanced |
| Namibia | Namibia Field Epidemiology Training Program | Advanced |
| Nigeria | Nigeria Field Epidemiology and Laboratory Training Program | Advanced |
| Pakistan | Pakistan Field Epidemiology and Laboratory Training Program | Advanced |
| Philippines | Philippines Field Epidemiology Training Program | Advanced |
| Singapore | Singapore Field Epidemiology Training Program | Advanced |
| Taiwan | Taiwan Field Epidemiology Training Program | Advanced |
| Thailand | Regional Field Epidemiology Training Program for Veterinarians | Intermediate; advanced |
| Turkey | Turkey Field Epidemiology Training Program | Advanced |
| UK | United Kingdom Field Epidemiology Training Program | Advanced |
| US CDC | Epidemic Intelligence Service | Advanced |
| Zambia | Zambia Field Epidemiology Training Program | Advanced |

* Besides curricula, several other documents were consulted: ECDC literature Review FETP Frontline Planning Guide, ECDC Technical Report, ECDC Competency document, WHO – ASPHER, One Health Core Competency Domains, e-Delphi process for environment health competencies (FTP-WEBE), Tripartite Guide to Addressing Zoonotic Diseases in Countries, Needs and Readiness Assessment Tool, Scoping Mission Tool, Frontline ISAVET curricula and training manuals for trainees, trainers, and mentors and Frontline ISAVET Monitoring and Evaluation Framework and Tools.

*Table S2. The number of One Health knowledge, skills and competency (KSC) statements in various domains and subdomains for frontline (F), intermediate (I), and advanced (A) training programmes in the final COHFE framework.*

| **Domains and subdomains** | **One Health (Core)** | | | |  | **One Health (Optional)** | | | |
| --- | --- | --- | --- | --- | --- | --- | --- | --- | --- |
|  | **F** | **I** | **A** | **T** |  | **F** | **I** | **A** | **T** |
| **Domain 1: Foundational knowledge and skills** | **6** | **10** | **8** | **24** |  | **6** | **10** | **5** | **21** |
| 1.1       History of epidemiology | 1 | 1 | 1 | 3 |  |  | 2 | 1 | 3 |
| 1.2 Epidemiology of infectious diseases | 3 | 3 |  | 6 |  | 3 | 2 | 1 | 6 |
| 1.3 Epidemiology of noncommunicable diseases | 1 | 1 | 2 | 4 |  |  |  |  |  |
| 1.4 Prioritization of disease and disease burden |  | 2 |  | 2 |  |  | 4 |  | 4 |
| 1.5 Policies and standards |  | 2 | 2 | 4 |  | 1 |  |  | 1 |
| 1.6 Maternal and child health: key indicators |  |  |  |  |  |  | 1 |  | 1 |
| 1.7 Demographic data and population dynamics |  |  |  |  |  | 1 | 1 | 2 | 4 |
| 1.8 Primary health services: key indicators |  |  | 1 | 1 |  |  |  |  |  |
| 1.9 Systems thinking | 1 | 1 | 2 | 4 |  | 1 |  | 1 | 2 |
|  |  |  |  |  |  |  |  |  |  |
| **Domain 2: Surveillance systems** | **10** | **8** | **10** | **28** |  | **15** | **4** | **7** | **26** |
| 2.1 Characteristics of a functional surveillance system | 1 | 1 | 1 | 3 |  | 7 | 1 | 1 | 9 |
| 2.2 Detection and reporting of cases, clusters, and health threats | 5 |  | 1 | 6 |  | 2 |  |  | 2 |
| 2.3 Surveillance data collection, analysis and interpretation | 2 | 1 | 1 | 4 |  | 2 | 1 | 2 | 5 |
| 2.4 Surveillance reporting | 1 | 2 |  | 3 |  | 3 | 1 | 1 | 5 |
| 2.5 Monitor and assess the quality of surveillance data | 1 | 2 |  | 3 |  |  |  |  |  |
| 2.6 Surveillance systems design and evaluation |  | 2 | 7 | 9 |  | 1 | 1 | 3 | 5 |
|  |  |  |  |  |  |  |  |  |  |
| **Domain 3: Field investigations** | **15** | **10** | **13** | **38** |  | **15** | **6** | **10** | **31** |
| 3.1 Field preparation | 5 | 4 | 5 | 14 |  | 5 | 3 | 4 | 12 |
| 3.2 Investigation | 6 | 2 | 2 | 10 |  | 7 | 1 |  | 8 |
| 3.3 Data management and analysis | 2 | 3 | 1 | 6 |  | 2 |  | 2 | 4 |
| 3.4 Reporting and follow-up interventions | 2 | 1 | 5 | 8 |  | 1 | 2 | 4 | 7 |
|  |  |  |  |  |  |  |  |  |  |
| **Domain 4: Disease management** | **19** | **10** | **18** | **47** |  | **8** | **9** | **12** | **29** |
| 4.1 Health systems and health service delivery | 2 |  | 1 | 3 |  | 1 | 1 | 4 | 6 |
| 4.2 Antimicrobial stewardship | 3 | 3 | 7 | 13 |  | 2 | 4 | 3 | 9 |
| 4.3 Immunizations | 8 | 3 | 4 | 15 |  | 2 | 1 | 2 | 5 |
| 4.4 Infectious diseases | 5 | 3 | 4 | 12 |  | 1 | 2 | 2 | 5 |
| 4.5 Disease management during travel, mobility, and movement | 1 | 1 | 2 | 4 |  | 2 | 1 | 1 | 4 |
|  |  |  |  |  |  |  |  |  |  |
| **Domain 5: Laboratory capacity** | **8** | **11** | **11** | **30** |  | **9** | **10** | **8** | **27** |
| 5.1 Necropsies, sample/specimen collection, labelling, storage, and transport | 3 | 3 | 2 | 8 |  | 1 | 2 | 2 | 5 |
| 5.2 Multisectoral planning and data linking | 1 | 2 | 3 | 6 |  | 2 | 4 | 5 | 11 |
| 5.3 Multisectoral coordination | 2 | 3 | 3 | 8 |  | 2 | 1 | 1 | 4 |
| 5.4 Analysis, interpretation and reporting of laboratory data | 2 | 3 | 3 | 8 |  | 4 | 3 |  | 7 |
|  |  |  |  |  |  |  |  |  |  |
| **Domain 6: Infection prevention and control, biosafety and biosecurity** | **15** | **12** | **11** | **38** |  | **12** | **5** | **10** | **27** |
| 6.1 Infection prevention and control, biosecurity and biosafety preparedness | 10 | 4 | 6 | 20 |  | 4 | 1 | 1 | 6 |
| 6.2 Infection prevention and control, biosecurity and biosafety implementation procedures | 3 | 5 | 3 | 11 |  | 5 | 3 | 5 | 13 |
| 6.3 Continuous quality improvement evaluation | 2 | 3 | 2 | 7 |  | 3 | 1 | 4 | 8 |
|  |  |  |  |  |  |  |  |  |  |
| **Domain 7: Preparedness and response** | **17** | **21** | **30** | **68** |  | **15** | **20** | **28** | **63** |
| 7.1 Detection of health threats | 3 | 3 | 8 | 14 |  | 4 | 2 | 2 | 8 |
| 7.2 Risk assessments | 1 | 3 | 4 | 8 |  | 4 | 4 | 1 | 9 |
| 7.3 Policy development, adaptation and implementation |  |  | 1 | 1 |  | 3 | 1 | 2 | 6 |
| 7.4 Preparedness and response planning | 3 | 5 | 6 | 14 |  | 2 | 6 | 6 | 14 |
| 7.5 Cross-sectoral coordination and incident management | 4 | 3 | 3 | 10 |  |  | 3 | 4 | 7 |
| 7.6 Emergency risk communication | 1 | 1 | 3 | 5 |  | 2 | 1 |  | 3 |
| 7.7 Mass gatherings | 3 | 3 | 1 | 7 |  |  |  | 5 | 5 |
| 7.8 Humanitarian crises and natural disasters | 1 | 2 | 2 | 5 |  |  | 2 | 6 | 8 |
| 7.9 Chemical, biological, radiological and nuclear emergencies | 1 | 1 | 2 | 4 |  |  | 1 | 2 | 3 |
|  |  |  |  |  |  |  |  |  |  |
| **Domain 8: Epidemiologic studies** | **10** | **10** | **21** | **41** |  | **13** | **9** | **8** | **30** |
| 8.1 Types of epidemiological studies | 2 | 2 |  | 4 |  | 4 | 2 | 3 | 9 |
| 8.2 Designing and planning epidemiological studies | 3 | 2 | 15 | 20 |  | 5 | 4 | 2 | 11 |
| 8.3 Conducting epidemiological field studies | 3 | 4 | 3 | 10 |  | 3 | 3 | 2 | 8 |
| 8.4 Reporting and publication of study findings | 2 | 2 | 3 | 7 |  | 1 |  | 1 | 2 |
|  |  |  |  |  |  |  |  |  |  |
| **Domain 9: Data management, biostatistics, and informatics** | **17** | **15** | **13** | **45** |  | **4** | **2** | **13** | **19** |
| 9.1 Planning for data collection and analysis | 3 | 2 | 3 | 8 |  | 1 |  |  | 1 |
| 9.2 Data collection | 3 | 4 | 5 | 12 |  | 3 |  | 4 | 7 |
| 9.3 Data analysis | 6 | 4 | 2 | 12 |  |  | 1 | 3 | 4 |
| 9.4 Data interpretation and presentation | 3 | 4 | 2 | 9 |  |  |  | 1 | 1 |
| 9.5 Digital tools | 2 | 1 | 1 | 4 |  |  | 1 | 5 | 6 |
|  |  |  |  |  |  |  |  |  |  |
| **Domain 10: Ecosystem health** | **11** | **7** | **12** | **30** |  | **25** | **24** | **33** | **82** |
| 10.1 Biodiversity and ecosystems | 2 | 1 | 3 | 6 |  | 7 | 12 | 11 | 30 |
| 10.2 Plant, animal and ecosystem health | 2 | 1 | 1 | 4 |  | 2 | 1 | 6 | 9 |
| 10.3 Air, water and soil quality | 2 | 1 | 2 | 5 |  | 6 | 2 | 3 | 11 |
| 10.4 Impacts of environmental degradation on health | 2 | 2 | 2 | 6 |  | 5 | 3 | 6 | 14 |
| 10.5 Anthropogenic, environmental and socioeconomic drivers of emerging health threats | 3 | 2 | 4 | 9 |  | 5 | 6 | 7 | 18 |
|  |  |  |  |  |  |  |  |  |  |
| **Domain 11: Leadership and management** | **7** | **9** | **13** | **29** |  | **31** | **21** | **15** | **67** |
| 11.1 Leadership and One Health | 2 | 2 | 2 | 6 |  | 9 | 3 | 2 | 14 |
| 11.2 Policy development and implementation | 1 | 1 | 2 | 4 |  | 5 | 4 | 5 | 14 |
| 11.3 Organizational management |  | 2 | 3 | 5 |  | 2 | 5 | 2 | 9 |
| 11.4 Project management | 1 | 2 | 4 | 7 |  | 2 | 3 | 1 | 6 |
| 11.5 Finance and budgeting |  | 1 | 2 | 3 |  | 7 | 3 | 2 | 12 |
| 11.6 Security in the field | 3 | 1 |  | 4 |  | 6 | 3 | 3 | 12 |
|  |  |  |  |  |  |  |  |  |  |
| **Domain 12: Communication and community engagement** | **5** | **5** | **8** | **18** |  | **10** | **11** | **14** | **35** |
| 12.1 Oral communication to technical and nontechnical audiences | 4 | 3 | 3 | 10 |  | 4 | 4 | 6 | 14 |
| 12.2 Written communication to technical and nontechnical audiences |  | 1 | 1 | 2 |  | 3 | 3 | 3 | 9 |
| 12.3 Risk communication | 1 |  | 1 | 2 |  |  | 2 | 5 | 7 |
| 12.4 Communication for events |  | 1 | 3 | 4 |  | 3 | 2 |  | 5 |
|  |  |  |  |  |  |  |  |  |  |
| **Domain 13: Training** | **1** |  | **3** | **4** |  | **24** | **25** | **22** | **71** |
| 13.1 Learning processes |  |  | 1 | 1 |  | 6 | 7 | 6 | 19 |
| 13.2 Learning needs assessment, training programme design, development and assessment | 1 |  | 2 | 3 |  | 5 | 6 | 3 | 14 |
| 13.3 Training delivery |  |  |  |  |  | 6 | 6 | 5 | 17 |
| 13.4 eLearning |  |  |  |  |  | 2 | 2 | 2 | 6 |
| 13.5 Quality and risk management in training |  |  |  |  |  | 5 | 4 | 6 | 15 |
|  |  |  |  |  |  |  |  |  |  |
| **Domain 14: Ethics** | **10** | **2** | **1** | **13** |  | **8** | **5** | **4** | **17** |
| 14.1 Ethics and its role related to health | 4 |  |  | 4 |  | 1 |  |  | 1 |
| 14.2 Ethical issues related to field epidemiology |  |  |  |  |  | 4 | 4 | 2 | 10 |
| 14.3 Moral challenges related to ethical decision-making | 2 | 1 | 1 | 4 |  | 2 | 1 | 2 | 5 |
| 14.4 Legal and regulatory ethical frameworks | 3 | 1 |  | 4 |  | 1 |  |  | 1 |
| 14.5 The five step process for ethical decision-making | 1 |  |  | 1 |  |  |  |  |  |
| **Grand Total** | **151** | **130** | **172** | **453** |  | **195** | **161** | **189** | **545** |

F: Frontline, I: Intermediate, A: Advanced, T: Total.
